# The synergistic effect of ribose, carnosine, and ascorbic acid on the sensory and physico-chemical characteristics of minced bison meat

**DOI:** 10.1002/fsn3.25

**Published:** 2013-02-14

**Authors:** Michel Aliani, Donna Ryland, Jennifer Williamson, Natalie Rempel

**Affiliations:** Department of Human Nutritional Sciences, University of ManitobaWinnipeg, Manitoba, Canada, R3T 2N2

**Keywords:** Ascorbic acid, bison patties, carnosine, physico-chemical analysis, ribose, sensory analysis

## Abstract

Ingredients such as ascorbic acid used to preserve redness of the raw meat, and carnosine and ribose used for flavor improvement have been incorporated into minced meats to increase consumer acceptance. The objective of this study was to investigate the possible synergistic effect of ascorbic acid, carnosine, and ribose on the sensory and physico-chemical characteristics of minced bison meat. Samples included control (Co) ±1% carnosine (C), 0.1% ascorbic acid (A), 2% ribose (R) (w/w), and combinations of RC, RA, RCA in the same concentrations as the single ingredient samples. A trained sensory panel (*n* = 7) measured the intensity of the aromas and flavors of salty, sour, beef, and liver of the bison patties. A consumer acceptance panel (*n* = 59) evaluated color, aroma, flavor, texture, and overall acceptability of the patties. Hunterlab colorimetry, shear force, cook loss, and drip loss percentage were measured on the cooked patties, and color and pH on the raw patties. The sample containing 2% ribose (R), 1% carnosine (C), and 0.1% ascorbic acid (A) in combination (RCA) showed a significantly higher consumer acceptance for aroma, which could possibly be attributed to the high beef aroma intensity measured by the descriptive analysis panel. RCA had the highest color acceptance which may be related to the high a* value for the cooked sample. RCA also had high overall acceptance corresponding to “like slightly.” Raw and cooked color values, shear force, pH, cook loss and drip loss percentages, and aroma and flavor attribute intensities for RCA were not significantly different from the control sample. The synergistic effect of ribose, carnosine, and ascorbic acid may positively affect the aroma and color of minced bison meat leading to higher overall acceptability without compromising sensory and physico-chemical quality.

## Practical Applications

Sensory and physico-chemical properties are of concern to the bison meat industry because they affect the consumer acceptability. Adding ingredients to minced bison such as ascorbic acid to improve redness of the raw meat, and carnosine and ribose for flavor is a strategy used to increase consumer acceptance of other red meats. The synergistic effect of a combination of these ingredients may be a way to improve the flavor and color of minced bison meat and thus increase consumer acceptability.

## Introduction

North American plains bison (*Bison bison bison*) are playing an increasing role in the livestock production of farmers in the United States and Canada. The combined herd size is estimated at 450,000 head (National Bison Association 2012). Consumer demand for bison meat is increasing due in part to its positive perception by health conscious consumers as being a more natural food product (Firmage-O'Brien 2008). It is a source of dietary red meat that contains 28.44 g protein, 2.4 g total fat, 3.4 mg iron, and 2.86 μg Vitamin B12 in 100 g of cooked bison roast (Health Canada 2010). In their review of the risks and benefits of red meat, McAfee et al. (2010) concluded that eating lean red meat in moderate amounts can lead to positive long-term health due to the nutrient content and fatty acid profile.

Taste/flavor of red meat received the highest score for importance (8.37 of 9) from 154 Canadian consumers surveyed about a number of different attributes related to the marketing of bison in a study conducted at the University of Saskatchewan (Sanderson et al. 2003). In this same study, 45% of the respondents agreed with the statement that bison meat has a “gamey” taste suggesting that improvement of flavor could possibly increase the consumer demand for bison meat.

Meat flavor precursors may be used to enhance the desirable flavor and aroma of cooked meats (Manley et al. 1999). Upon cooking, reducing sugars present in meat can react with free amino acids via the Maillard reaction to generate hundreds of volatile compounds affecting the overall aroma and flavor of cooked meat (Mottram 1991). Ribose was shown to increase the “roasted” and “chicken” odors of cooked minced chicken breast meat when added in an amount equal to two- to fourfold its natural concentrations (Aliani and Farmer 2005).

Carnosine, a natural occurring dipeptide found in meat, changed the distribution of volatiles when added to oxidized liposomes which could lead to altered flavor properties (Kansci et al. 1997). Carnosine added to a model system which included ribose and cysteine in equimolar concentrations was suggested as playing a role in the production of roasty volatiles (Chen and Ho 2002). These changes in flavor could be due to the ability of carnosine to act as an antioxidant by inhibiting the formation of lipid peroxides and thiobarbituric acid–reactive substances leading to lower sensory oxidative rancidity (Decker and Crum 1991). This capability is especially important for minced meat as according to Sato and Hegarty (1971) oxidized flavor can occur within 1 h of grinding. Minced buffalo meat containing 0.5% carnosine showed a significantly higher pleasant odor in raw minced meat compared with the control after 10 days of refrigerated storage.

The presence of bright red color of fresh meat is also important to consumers. Carnosine (0.5%) added to buffalo meat resulted in significantly higher red color both visually and instrumentally over the 10-day storage period compared with the control (Das et al. 2006). Lee et al. (1999) found that compared with the control, the addition of both ascorbic acid and carnosine successfully increased the amount of red color in ground beef. They suggested that this may be due to carnosine protecting ascorbic acid against oxidation caused by metal chelators found in meat.

Therefore, there is some evidence to suggest that ribose, carnosine, and ascorbic acid added to meat can positively affect the odor and hence the flavor as well as color. However, studies are lacking regarding the effect of these additives on bison meat. Thus, the objective of this study was to investigate the synergistic effect of added ribose, carnosine, and ascorbic acid on the flavor, acceptability, and physico-chemical properties of minced bison meat.

## Materials and Methods

### Meat

North American bison (*Bison bison bison*) animals, one heifer and three males, were born in May or June 2009 and were slaughtered on 9 February 2011. Animals (approximately 8–13 months old) were fed free-choice hay of high-quality grass and kept in a 1 acre pen. Free-choice hay of medium-quality grass was fed for the last 150 days. Animals were on free-choice water (deep well). The supplement ration was peas and lentil screenings with 10% added wheat screenings, chelated minerals for copper, zinc, and manganese, Vitamin A (1 million IU) and Vitamin E (1500 IU). Mineral was also available in the free-choice diet the same as in the supplement. Supplement consumption was estimated between 14 and 16 pounds per day from a self-feeder. All animals had more than 60 days since the last Ivomec treatment (withdrawal for Ivomec is 42 days). Slaughter was conducted at a provincially inspected facility, OakRidge Meats, McCreary MB, according to the Canadian Food Inspection Agency (CFIA) regulations.

### Ingredients

Ribose (ProFood International, Inc. Lisle, IL), ascorbic acid (Natural Factors, Coquitlam, British Columbia, Canada), and carnosine (Young Again Products, Wilmington, NC) were all food grade.

### Processing

Processing took place in the same facility as for slaughter. Bison was made into 95% lean ground meat using the chuck and round (50/50) taking a homogenous sample from the four animals. Meat was minced using a 3 mm grind size for the finished product. Minced bison was vacuum packaged into 2-kg lots, refrigerated, and then frozen at −20°C for 13 days after slaughter.

### Patty making

The 2-kg package was thawed in cold water for approximately 2 h. Seven treatments were prepared: control (Co), ribose (R) (2%), carnosine (C) (1%), ascorbic acid (A) (0.1%), and combinations of ribose and carnosine (RC), ribose and ascorbic acid (RA), and ribose, carnosine, and ascorbic acid (RCA) in the same concentrations as individual addition. The method for incorporating the solutions into meat was as follows. Meat was preblended for 5 sec on low using a handheld mixer (KitchenAid, St. Joseph, MI). Liquid was added in three lots with a 5-sec mix between and 10 sec at the end for a total mix time of approximately 30 sec. Minced meat (130 g) was formed into a uniform shape about 7 cm diameter and 4 cm high and placed in the center of the hamburger stacker (Hamburger Stacker Starfrit Brand made in China for Atlantic Promotions Inc., Mississauga, Ontario, Canada). The press was placed on a plate on top of the meat and was uniformly pushed down checking that the thickness of the patty was the same all around. Final patty dimensions were approximately 11 cm diameter × 1 cm thick. Patties were wrapped in plastic, placed in a sealed plastic container (Ziploc imported by S. C. Johnson and Son, Limited, Brantford, Ontario, Canada), and stored for approximately 18 h at 4°C.

### Sample preparation for sensory evaluation

Conventional residential ovens (Frigidaire Electric Range, ES510 Control, Electrolux Canada Corp., Mississauga, Ontario, Canada) were preheated on the broil setting to 260°C for at least 20 min. Patty samples were placed on racks that were set into a baking sheet (45 × 28 × 2 cm) covered with foil wrap. Pans were 7 cm from the heating element. Patties were turned once when the internal temperature was approximately 66°C and removed from the oven when the final internal temperature was 72–73°C as read by a Traceable Full-Scale Plus digital thermometer (Control Company, Friendswood, TX). Total cook time was approximately 12 min. Cooked patty dimensions were approximately 8.5 cm diameter × 1 cm thick. Patties were cut into six pie-shaped pieces (approximately 10 g each), wrapped in foil, and placed in Styrofoam containers to keep hot. For serving, each piece was placed into a labeled 96.1 mL plastic portion cup (Solo Cup Company, Lake Forest, IL) and capped just prior to evaluation by the panelists.

### Sensory methods

#### Recruitment

Volunteers were recruited from the staff and student populations according to procedures approved by the Human Ethics Research Board at the University of Manitoba for both the descriptive analysis and consumer acceptance studies. The only criteria for panelists were that they be available, interested in participating, and were not allergic to any food products. For descriptive analysis, the panelists consisted of four males and three females between 22 and 35 years of age. An honorarium was provided to participants. For consumer acceptance, 44 female and 15 male respondents over 18 years of age completed questionnaires receiving a snack bar or juice box for their participation.

#### Descriptive analysis

Sensory attribute intensities were measured using a modified quantitative descriptive analysis sensory method (Stone and Sidel 2004). Training was conducted by an experienced group leader during six sessions of 45 min each. The aroma and flavor attribute descriptors of the minced bison samples coded with three-digit random numbers were noted individually and then discussed during the first session. From these descriptors, standard products were selected that represented the definition of the attributes and their intensities were measured on the 15-cm unstructured line scale from 0 (labeled low) to 15 (labeled high) (Table 1). Four aroma, two taste, and two flavor attributes were used to describe the different bison samples. During the remaining sessions, the aroma and flavor attribute intensities of the seven samples were evaluated repeatedly by all panelists. Discussion of the results followed so that panelists could become more consistent within their own measurements of the attributes as well as within the measurements of the group members. One-way analysis of variance (ANOVA) was conducted to determine the reproducibility of panelist measurements as recommended by Stone and Sidel (2004).

**Table 1:** Sensory attribute definitions, method for meat evaluation, point on line scale, standard preparation, and manufacturer

Attribute	Definition	Method for meat evaluation	Point on 15-cm line scale	Standard preparation	Manufacturer
Aroma					
Salty	Aroma associated with beef broth powder	Place the sample container in position for sniffing. Remove the cover. Take three short sniffs and replace the cover	9.4	Beef OXO powder (4.5 g) dissolved in boiling water (175 g) (according to directions), let stand approximately 20 min before serving (20 mL in 60 mL portion cup)	Knorr, Unilever Canada, Toronto, Ontario, Canada
Sour	Aroma associated with vinegar	Place the sample container in position for sniffing. Remove the cover. Take three short sniffs and replace the cover	12.0	White vinegar, 4–5% acetic acid (30 mL amber vial with three drops vinegar on cotton ball)	H. J. Heinz Company of Canada Ltd., North York, Ontario, Canada
Beef	Aroma associated with cooked minced beef	Place the sample container in position for sniffing. Remove the cover. Take three short sniffs and replace the cover	10.6	Lean (17% fat) ground beef patty broiled in preheated oven (230°C) to 72–73°C (1/6 patty in 96.1 mL portion cup)	Safeway Inc., local supermarket, Winnipeg, Manitoba, Canada
Liver	Aroma associated with cooked beef liver	Place the sample container in position for sniffing. Remove the cover. Take three short sniffs and replace the cover	10.9	5% raw pureed beef liver incorporated into lean ground bison, formed into patty (130 g) and cooked as for beef aroma standard.(1/6 patty in 96.1 mL portion cup)	Safeway Inc., local supermarket, Winnipeg, Manitoba, Canada
Taste/Flavor					
Salty	Taste associated with sodium chloride in water	Take one sip of the sample. Evaluate the taste attribute intensity just before swallowing the sample	8.5	0.4% (wt/vol) sodium chloride (0.4 g sodium chloride in 100 mL filtered water) (20 mL in 60 mL portion cup)	Coarse salt; Sifto Canada Inc., Mississauga, Ontario, Canada
Sour	Taste associated with citric acid in water	Take one sip of the sample. Evaluate the taste attribute intensity just before swallowing the sample	4.6	0.04% (wt/vol) citric acid (0.04 g citric acid in 100 mL filtered water) (20 mL in 60 mL portion cup)	Rougier Pharma, Division of Ratiopharm Inc., Mirabel, Canada
Beef	Flavor associated with cooked minced beef	Take one bite of the sample. Chew it thoroughly. Evaluate the flavor attribute intensity just before swallowing the sample	9.1	Lean (17% fat) ground beef patty broiled in preheated oven (230°C) to 72–73°C (1/6 patty in 96.1 mL portion cup)	Safeway Inc., local supermarket, Winnipeg, Manitoba, Canada
Liver	Flavor associated with cooked liver	Take one bite of the sample. Chew it thoroughly. Evaluate the flavor attribute intensity just before swallowing the sample	10.3	5% raw pureed beef liver incorporated into lean minced bison, formed into patty (130 g) and cooked as for beef aroma standard (1/6 patty in 96.1 mL portion cup)	Safeway Inc., local supermarket, Winnipeg, Manitoba, Canada

For the experimental sessions, panelists were seated in individual partitioned work stations equipped with computerized sensory software (Sensory Information Management System 2011). Light from incandescent bulbs directed through red opaque plastic was used to mask possible color differences between the samples. Filtered water and unsalted topped crackers were available for cleansing the palate as required. All of the panelists received samples from the seven minced bison formulations coded with three-digit random numbers presented according to the randomized plan generated by the sensory software. For each sample, panelists were instructed to smell the sample and mark intensities for all of the aroma attributes followed by tasting the sample and marking intensities for all of the taste/flavor attributes. Samples were evaluated by all panelists on three separate days within a 1-week period to make three replications.

#### Consumer acceptance

Consumers were seated at the same work stations equipped with the same computerized sensory software as for the descriptive analysis study. Overhead fluorescent light was used. Filtered water was available for cleansing the palate as required. All consumers evaluated the seven minced bison samples all at once during one session. Samples were presented as for descriptive analysis. After smelling, observing, and tasting as much of the sample as desired, consumers rated the aroma, color, flavor, and texture as well as overall acceptance of the meat samples on 9-point hedonic scales (Stone and Sidel 2004). The food action rating scale (FACT) was used as another measure of acceptance based on how often consumers would eat the minced bison samples that they tasted (Schutz 1965). One of the following nine categories could be selected where 9 = I would eat this every opportunity I had; 8 = I would eat this very often; 7 = I would frequently eat this; 6 = I like this and would eat it now and then; 5 = I would eat this if available but would not go out of my way; 4 = I do not like this but would eat it on an occasion; 3 = I would hardly ever eat this; 2 = I would eat this if there were no other food choices; 1 = I would eat this only if forced. Information was collected regarding gender, age, and frequency of eating burgers of any type.

### Physical and chemical measurements

#### Instrumental color

The Hunterlab Miniscan (Hunter Associates Laboratory, Inc., Reston, VA) with illuminant D65 and 10° standard observer angle standardized to the white tile was used to measure L*, a*, and b* values. Raw meat patties were removed from the refrigerator, uncovered, and allowed to “bloom” for 30 min. Cooked patties were measured at room temperature. Three readings were taken on different portions of the patty surface which was covered with a glass plate. Two patties from each of three batches were measured.

#### Shear force

Patties were cooked using the same method as those for the sensory evaluation and cooled to room temperature. Oval edges were cut from each patty to make it square. Four pieces were cut from each patty (approximately 1 cm wide, 1 cm high, and 5 cm long). Meat pieces were placed one at a time into the V-shaped Warner Bratzler attachment mounted on the Lloyd Texture Measuring Instrument (Lloyd Instrument, L1000R, Lab Integration, Mississauga, Ontario, Canada) with the last side cooked facing up, and sheared at 200 mm/min. Newtons of force required to shear the piece were recorded. Four patties from each of four batches were measured.

#### Cooking losses

The cooking loss was calculated as: ([*W*_r_ − *W*_c_]/*W*_r_) × 100, where *W*_r_ was the weight of the raw patty and *W*_c_ was the weight of the cooked patty (Ganhão et al. 2010). Liquid remaining on the foil was cooled to room temperature and was weighed to represent drip loss (DL). Drip loss percentage was calculated as DL/*W*_c_ × 100, where *W*_c_ was the cooked patty weight. Two patties from each of four batches were measured.

#### pH

The adapted method of Troutt et al. (1992) was employed where 10 g of raw meat was placed in 100 g filtered water in a mini food processor bowl (Cuisinart Canada, Woodbridge, Ontario, Canada) and processed for 1 min. Fat fibers were strained and the sample was stirred immediately prior to pH measurement (Oakton Model 35624-35, Oakton Instruments, Vernon Hills, IL). Measurements were made in triplicate.

### Statistical analysis

For descriptive analysis, three-way ANOVA used the model that included *panelist* (P) and *replication* (R) as random effects and *formulation* (F) as the fixed effect. All two-way interactions *panelist by replication*, *panelist by formulation*, and *replication by formulation* were analyzed. For consumer acceptability, four-way ANOVA was conducted with *consumer* (C) as a random effect and *formulation* (F), *gender* (G), and *age group* (A) as fixed effects. The two-way interaction of *gender by age group* was analyzed. For all other measurements except for pH, two-way ANOVA was performed with *formulation* and *patty* as fixed effects including analysis of the *formulation by patty* interaction. One-way ANOVA was used for pH analysis. When interactions were not significant, they were pooled with the error (O'Mahony 1986). *F*-values were recalculated with the additional sums of squares for error and the corresponding degrees of freedom. Tukey's multiple comparison test was used to determine the mean treatment differences when significant (*P* < 0.05). To relate the seven formulations of minced bison to flavor and aroma attribute intensities, principal component analysis was performed using the average values from each sample. For the consumer acceptability data, a principal component model was fit where the value for overall acceptability for each consumer was plotted, with consumers represented as vectors and samples represented by points on the graph. Stepwise multiple regression analysis was performed as recommended by MacFie and Thomson (1988) to determine the influence of measured sensory attribute intensities, physical and chemical measurements on aroma acceptance. SAS Statistical System (2003) software (Statistical Analysis System, Cary, NC) was used for all of the above analyses except for stepwise multiple regression which used SPSS (2010).

Partial least squares regression was conducted using mean values to test if the sensory, physical, and chemical measurements discriminated the seven bison samples and to visualize the separation of the groups on a two-dimensional biplot (XLSTAT 2012).

## Results and Discussion

Concentrations of additives were selected based on work from previous researchers. Das et al. (2006) found that 1% carnosine added to ground buffalo meat helped to maintain fresh meat color and desirable odor for at least 8 days at 4°C. Lee et al. (1999) added 0.1% ascorbic acid and 1% carnosine to ground beef, and concluded that the synergistic effect may prove beneficial for eliminating off-flavor formation and increasing shelf life. An addition of 1% ribose to chicken breast appeared to increase chicken, meaty, and roasted aromas and decrease off-odors (Aliani and Farmer 2005). An additional 1% was added in this study to compensate for the other additives possibly affecting the flavor.

### Physical and chemical measurements

#### Instrumental color

Among the additives used, ascorbic acid was expected to have the most influence on color of raw samples, and ribose the most influence on cooked color. Color measurements taken with the CIELab system provide visual hue values where a higher a* value indicates higher red color and a higher b* value indicates higher yellow color. Higher L* value indicates a lighter sample. For raw patties, the ribose sample showed significantly lower red color than all of the other samples (Table 2). Compared with beef containing 5% fat (Troutt et al. 1992), the a* values showed lower redness for minced bison (approximately 13 vs. 20) which may contribute to a darker meat. This finding is in agreement with Joseph et al. (2010) who found that bison meat was visually darker than beef. For the cooked samples, RCA was significantly higher in redness (a* value) than Co, C, and A samples but not significantly different from the other samples containing ribose (Table 2). The R sample had the highest yellowness (b* value) which was significantly higher than the sample containing A. The addition of ribose appeared to influence the cooked color of the patties possibly as a result of the Maillard reaction.

**Table 2:** *F*-value with associated probabilities and mean value (standard error of the mean) from chemical and physical testing of seven formulations of minced bison meat (F = formulation; P = patty)

	Source of variation (*F*-value)			Mean value for formulation (*n* = 7)						
		
	F	P	F × P	Co	R	C	A	RA	RC	RCA
Raw color (*n* = 6)										
L*	1.09 NS	0.62 NS	0.38 NS	36.4^a^ (0.4)	36.0^a^ (0.3)	35.3^a^ (0.4)	36.1^a^ (0.3)	35.7^a^ (0.3)	35.8^a^ (0.2)	35.8^a^ (0.2)
a*	4.92**	0.31 NS	0.85 NS	13.0^a^ (0.1)	11.9^b^ (0.1)	13.1^a^ (0.3)	13.1^a^ (0.1)	13.0^a^ (0.2)	13.0^a^ (0.2)	13.6^a^ (0.4)
b*	1.96 NS	0.53 NS	1.59 NS	16.2^a^ (0.2)	15.7^a^ (0.2)	15.7^a^ (0.3)	15.9^a^ (0.2)	16.1^a^ (0.1)	15.9^a^ (0.1)	16.5^a^ (0.2)
Cooked color (*n* = 6)										
L*	1.03 NS	2.09 NS	0.83 NS	33.4^a^ (0.7)	33.2^a^ (0.6)	33.4^a^ (0.5)	33.6^a^ (0.7)	32.5^a^ (1.0)	32.8^a^ (0.9)	31.2^a^ (1.1)
a*	7.72***	2.17 NS	2.91*	5.8^bc^ (0.1)	6.4^abc^ (0.2)	5.7^c^ (0.1)	5.7^c^ (0.1)	6.63^a^ (0.3)	6.58^ab^ (0.3)	6.9^a^ (0.2)
b*	3.41**	0.00 NS	2.05 NS	14.6^ab^ (0.3)	16.0^a^ (0.4)	14.4^ab^ (0.3)	13.4^b^ (0.6)	14.8^ab^ (0.4)	14.3^ab^ (0.4)	14.1^ab^ (0.5)
Shear force (*N*) (*n* = 16)	0.52 NS	5.90**	2.04*	13.31^a^ (0.77)	12.03^a^ (1.37)	12.60^a^ (1.40)	11.25^a^ (1.10)	12.38^a^ (0.86)	11.64^a^ (1.23)	12.69^a^ (0.77)
Cooking loss percentage (*n* = 8)	1.84 NS	0.89 NS	0.34 NS	43.67^a^ (0.54)	44.54^a^ (0.55)	45.67^a^ (0.74)	44.23^a^ (0.54)	45.43^a^ (0.86)	46.25^a^ (0.86)	45.62^a^ (0.59)
Drip loss percentage (*n* = 8)	2.83*	5.00*	0.34 NS	17.90^ab^ (0.92)	18.04^ab^ (1.70)	16.38^ab^ (0.87)	14.73^b^ (0.88)	16.59^ab^ (0.98)	20.62^a^ (1.33)	18.26^ab^ (1.06)
pH (*n* = 3)	19.74***			5.75^bc^ (0.02)	5.72^bc^ (0.01)	5.89^a^ (0.02)	5.68^c^ (0.01)	5.72^bc^ (0.01)	5.79^b^ (0.00)	5.75^bc^ (0.01)

Co, control; R, ribose; C, carnosine; A, ascorbic acid; RA, ribose + ascorbic acid; RC, ribose + carnosine; RCA, ribose + carnosine + ascorbic acid. Means with the same letter within the same row are not significantly different with a significance level of *P* < 0.05.

NS: *P* ≥ 0.05,

* *P* < 0.05,

** *P* < 0.01,

*** *P* < 0.001.

#### pH

Solutions of additives were measured for pH with the following results – 8.31 for carnosine (1%), 8.01 for ribose (2%), and 3.84 for ascorbic acid (0.1%). Perceived tastes of the solutions were evaluated by the researchers. As expected, carnosine was very slightly bitter, ribose was slightly sweet, and ascorbic acid was slightly sour. Values for pH for bison samples ranged from 5.68 for A to 5.89 for C with the control closer to the lower end at 5.75 (Table 2). Even with the additives, the pH compared favorably with the results reported by Li and Logue (2005) who found that the original pH of bison meat ranged from 5.63 to 5.72. The sample containing carnosine had significantly higher pH than all of the other samples (Table 2) likely due to its high initial pH in solution as noted above. Ribose in addition to carnosine produced a patty with significantly lower pH than when carnosine was added alone which may be beneficial for delay of microbial deterioration (Table 2).

#### Cooking losses

The cooking loss percentage has been shown to be related to pH where an increasing pH resulted in a decreased cooking loss (Lee et al. 1999; Das et al. 2006). In this study, there were no significant differences shown for cooking loss even though the pH for C was significantly higher than that for A (Table 2). This disagreement may be due to the method used for cooking loss. A dry heat method was used in our study compared with a water bath immersion of the sample in an enclosed bag used by the previous researchers. The higher pH of their 1% carnosine sample (6.02 vs. 5.89) also may have affected this result. Bison patties cooked by the broiling method to 71°C had a cooking yield of 74.2% (Yuan et al. 1999), which was higher than that observed in this study (approximately 56%). This could be due to the higher internal cooking temperature (72–73°C vs. 71°C) and longer cooking time (approximately 12 min vs. 10 min) in addition to the hand pressing versus mechanical pressing of the patties in this study. DL percentage, a measurement of the combination of fat and moisture emitted from the patty during cooking, was significantly lower for sample A compared with the RC sample. Sample A also had a significantly lower pH than the RC sample.

#### Shear force

Shear force measurements showed no significant differences between the seven bison formulations (Table 2). The control sample had the highest shear force and the ascorbic acid sample the lowest. This may be explained partially by low DL percentage of the ascorbic acid sample (Table 2) resulting in higher moisture retention leading to a softer product.

#### Sensory evaluation

For descriptive analysis, panelists were not screened or selected for any sensitivities and thus panelists were considered a random effect. Contributing to this consideration is the fact that panelists are inherently variable (Lundahl and McDaniel 1988). Analysis of panelists as a random effect allows for the conclusions drawn to be extended to the general population (O'Mahony 1986). This reasoning is also applied to the replications of panelist evaluations on three separate days where conclusions drawn could be extended to all replications of minced bison samples conducted. Formulation of minced bison samples is considered a fixed effect as the conclusions drawn would only be applicable to the samples as they were prepared for this study. It should also be noted that with a random panelist effect, the ability to show significant differences in treatments is decreased due to the reduction in the degrees of freedom for this source of variation. With this reduction in degrees of freedom, a larger F-ratio is required in order to show a significant difference (Lundahl and McDaniel 1988). For consumer acceptance, panelists are random effects as by definition they must be representative of the consumer population (Lundahl and McDaniel 1988).

#### Descriptive analysis

Replication was consistent throughout the 3 days of evaluation as no attributes showed a significant difference between replications (Table 3). Only sour taste showed a significant replication by formulation interaction (*P* < 0.05).

**Table 3:** *F*-value with associated probabilities and mean value (standard error of the mean) for descriptive analysis of formulations of minced bison meat from three-way ANOVA (R = replicate [*n* = 3]; P = panelist [*n* = 7]; F = formulation [*n* = 7])

	Source of variation (*F*-value)						Mean intensity value (*n* = 21) for formulation (0 = low; 15 = high)						
		
Attribute	R	P	F	R × P	R × F	P × F	Co	R	C	A	RA	RC	RCA
Aroma													
Salty (Salty_AR)	0.92 NS	19.69***	0.19 NS	0.85 NS	0.71 NS	0.42 NS	5.4 (0.7)	5.1 (0.6)	5.3 (0.6)	5.6 (0.7)	5.2 (0.6)	5.0 (0.5)	5.1 (0.6)
Sour (Sour_AR)	2.75 NS	33.30***	1.43 NS	0.85 NS	0.91 NS	0.96 NS	3.6 (0.5)	3.7 (0.3)	3.7 (0.5)	3.5 (0.5)	4.5 (0.7)	4.3 (0.6)	3.8 (0.5)
Beef (Beef_AR)	0.53 NS	28.19***	0.86 NS	0.96 NS	0.74 NS	0.46 NS	7.5 (0.7)	7.3 (0.7)	7.4 (0.6)	7.8 (0.7)	8.0 (0.7)	8.0 (0.6)	8.5 (0.6)
Liver (Liver_AR)	0.28 NS	11.54***	0.90 NS	2.69**	1.54 NS	1.03 NS	5.5 (0.7)	6.7 (0.7)	6.2 (0.6)	5.7 (0.6)	6.0 (0.8)	5.6 (0.6)	5.8 (0.7)
Taste/Flavor													
Salty (Salty_TA)	0.10 NS	6.65**	1.25 NS	4.32***	1.36 NS	1.30 NS	4.7 (0.7)	5.1 (0.7)	4.3 (0.6)	3.9 (0.5)	5.0 (0.6)	4.6 (0.5)	4.2 (0.6)
Sour (Sour_TA)	2.01 NS	14.86***	1.24 NS	6.87***	2.01*	1.67*	4.2 (0.6)	3.9 (0.6)	4.0 (0.6)	3.8 (0.5)	4.8 (0.7)	4.0 (0.6)	3.5 (0.4)
Beef (Beef_FL)	0.47 NS	24.27***	0.50 NS	2.06*	1.68 NS	2.08**	8.9 (0.6)	8.5 (0.7)	8.7 (0.6)	8.0 (0.7)	8.7 (0.6)	8.6 (0.6)	8.3 (0.6)
Liver (Liver_FL)	0.79 NS	119.72***	0.70 NS	1.85 NS	0.77 NS	0.91 NS	5.2 (0.7)	4.9 (0.7)	5.2 (0.7)	5.6 (0.8)	5.4 (0.7)	5.2 (0.7)	5.0 (0.7)

ANOVA, analysis of variance; Co, control; R, ribose; C, carnosine; A, ascorbic acid; RA, ribose + ascorbic acid; RC, ribose + carnosine; RCA, ribose + carnosine + ascorbic acid.

NS: *P* ≥ 0.05,

* *P* < 0.05,

** *P* < 0.01,

*** *P* < 0.001.

Panelists were shown to be significantly different for all attributes (*P* < 0.001 except for salty taste *P* < 0.01) which is not unexpected (Table 3). Even though training helps to decrease the variability among panelists due to different sensitivities and use of the line scale, it cannot eliminate it (Lundahl and McDaniel 1988). A significant interaction between panelists and formulation indicates that all panelists are not evaluating the samples in the same order or that the magnitude of the sample intensities differs between panelists. This was shown for sour taste (*P* < 0.05) and beef flavor (*P* < 0.01). Significant interaction for panelist by replication indicates inconsistency for panelists throughout the replications and was shown for liver aroma (*P* < 0.01), salty (*P* < 0.001) and sour (*P* < 0.001) tastes, and beef flavor (*P* < 0.05). Investigation of the interaction plots determined that no single panelist was consistently contributing to the significant interactions.

It should be noted that throughout the training period, attributes were developed and reinforced with repeated evaluations to ensure that no additional attributes especially related to oxidation were detected. Meat flavor is a very complex field of study (Calkins and Hodgen 2007) as meat contains many water- and fat-soluble compounds. As noted by MacLeod (1994), water-soluble compounds alone contribute to the sweetness, sourness, bitterness, saltiness, and umami tastes of beef by way of sugars, organic acids, peptides, inorganic salts, and the salts of amino acids, respectively. No significant differences were found among the control and the six formulations for any of the four aromas, two taste, and two flavor attribute intensities measured (Table 3). Therefore, it appeared that the levels of additives used in the formulations did not alter the bison patties significantly compared with the control. In order to see a significant effect of these additives, it may be advised to increase the concentrations. Including glucose as one of the additives may be another way to impact flavor as Meinert et al. (2009) concluded that for pork, glucose exerted a greater influence on the concentration of volatiles compared with ribose.

The formulations with the highest mean aroma intensity values were A for salty aroma, RA for sour aroma, RCA for beef aroma, and R for liver aroma. For flavor intensities, the formulations with the highest mean values were R for salty, RA for sour, Co for beef, and A for liver. McClenahan et al. (2001) used a trained panel to evaluate the overall aroma and flavor intensity of bison patties that were grilled and broiled. For both methods, which showed no significant differences, the samples obtained scores of approximately 3.3 and 4.0 on a 6-point scale ranging from very weak aroma/very bland flavor to very strong aroma and very intense flavor. In our study, beef aroma (8.5 for RCA) and beef flavor (8.9 for Co) were found to have the highest intensities compared with the other attributes which ranged from 4 to 6 on the 15-cm line scale (Table 3). Descriptions of the flavor and aroma attributes in this study will help to understand how the additives are affecting the meat and their influence on consumer acceptability.

**Figure 1:**
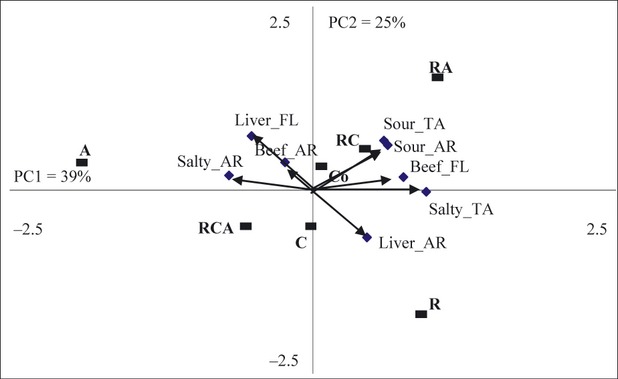
Biplot of factor 1 (*x*-axis) and factor 2 (*y*-axis) for sensory attributes (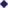) by trained panel with seven minced bison formulations (▪). Co, control; R, ribose; C, carnosine; A, ascorbic acid; RA, ribose + ascorbic acid; RC, ribose + carnosine; RCA, ribose + carnosine + ascorbic acid.

Principal component analysis determined that 86% of the variability in the experiment was attributed to the first three principal components. Based on the high factor loadings (absolute values over 0.6), the first component (39% of the variability) was comprised of salty and sour aromas, salty taste, and beef flavor. The second component (25% of the variability) was comprised of liver aroma, sour taste, and liver flavor. The third component (22% of the variability) was composed of beef aroma. The loadings from the attributes and the seven bison formulations for factors 1 and 2 were plotted and are shown in Figure 1. Samples including R, RA, RC, and Co appeared in the right half of the plot with sour aroma and taste, beef flavor, salty taste, and liver aroma. Liver flavor, salty and beef aromas shared the left half with A, RCA, and C.

### Consumer acceptability

#### Demographics

Of the 59 consumers in the study, 75% were female and 64% were less than 35 years of age. Forty-six percent of the consumers ate burgers “at least once a month,” 27% “at least once a week,” 7% “two to three times a week,” and 8% “more than three times per week.” The remaining 12% ate burgers “occasionally.”

#### Sample evaluation

A significant difference was shown for the main effect of consumer for all attributes (*P* < 0.001) which was to be expected as everyone has their own acceptance criteria, methods for using the category scales, and sensitivities to the ingredients present in the minced bison (Table 4). There was no significant difference shown between genders for any of the acceptance parameters. Mean values for acceptance of color and flavor were significantly higher (*P* < 0.05) for those in the older age group. Aroma was the only attribute which showed a significant difference among the seven samples with RCA showing a significantly higher acceptance (7.0 – like moderately) for aroma compared with Co (6.3), RA (6.4), and C (6.5). RCA also had the highest number of responses in the “like very much” category (44%) compared with the next highest sample RC which had 31% in that category. Co had 20% of responses in the “like very much” category. Color acceptance of the RCA sample was the highest of the seven samples and may be related to its significantly higher instrumental redness value for the cooked color compared with the control. This sample also had the highest redness value for raw meat.

**Table 4:** *F*-value with associated probabilities and mean value (standard error of the mean) for consumer acceptance of minced bison from four-way ANOVA (C = consumer [*n* = 59]; G = gender [*n* = 2]; A = age group [*n* = 2]; F = formulation [*n* = 7])

	Source of variation (*F*-value)					Mean value for gender		Mean value for age group		Mean value (*n* = 59) for formulation						
				
Attribute	C	G	A	F	G × A	Female (*n* = 44)	Male (*n* = 15)	16–34 years (*n* = 38)	Over 34 years (*n* = 21)	Co	R	C	A	RA	RC	RCA
Aroma^1^ (Aroma_Accept)	6.11***	1.44 NS	0.31 NS	2.75*	0.00 NS	6.7^a^ (0.1)	6.4^a^ (0.2)	6.6^a^ (0.1)	6.7^a^ (0.1)	6.3^c^ (0.2)	6.8^ab^ (0.2)	6.5^bc^ (0.2)	6.6^abc^ (0.2)	6.4^bc^ (0.2)	6.9^a^ (0.2)	7.0^a^ (0.2)
Color^1^ (Color_Accept)	5.45***	0.24 NS	4.71*	1.42 NS	0.06 NS	6.7^a^ (0.1)	6.6^a^ (0.1)	6.5^b^ (0.1)	7.0^a^ (0.1)	6.4^a^ (0.2)	6.6^a^ (0.2)	6.7^a^ (0.2)	6.6^a^ (0.2)	6.6^a^ (0.2)	6.7^a^ (0.2)	6.9^a^ (0.2)
Flavor^1^ (Flavor_Accept)	3.26***	0.56 NS	6.37*	0.40 NS	0.20 NS	6.3^a^ (0.1)	6.1^a^ (0.2)	6.0^b^ (0.1)	6.7^a^ (0.1)	6.2^a^ (0.2)	6.4^a^ (0.2)	6.3^a^ (0.2)	6.1^a^ (0.2)	6.3^a^ (0.2)	6.3^a^ (0.2)	6.3^a^ (0.2)
Texture^1^ (Texture_Accept)	3.48***	0.10 NS	1.39 NS	1.02 NS	0.05 NS	5.9^a^ (0.1)	6.0^a^ (0.2)	5.8^a^ (0.1)	6.2^a^ (0.1)	5.7^a^ (0.2)	5.8^a^ (0.2)	6.2^a^ (0.2)	5.7^a^ (0.2)	6.1^a^ (0.2)	6.0^a^ (0.3)	6.1^a^ (0.2)
Overall acceptability^1^ (Overall_Accept)	3.44***	0.13 NS	2.57 NS	0.79 NS	0.26 NS	6.2^a^ (0.1)	6.1^a^ (0.2)	6.0^a^ (0.1)	6.4^a^ (0.1)	6.0^a^ (0.2)	6.3^a^ (0.2)	6.2^a^ (0.2)	5.9^a^ (0.3)	6.1^a^ (0.2)	6.2^a^ (0.2)	6.3^a^ (0.2)
FACT^2^	4.94***	0.05 NS	0.45 NS	1.19 NS	1.96 NS	5.3^a^ (0.1)	5.3^a^ (0.2)	5.2^a^ (0.1)	5.4^a^ (0.1)	5.1^a^ (0.2)	5.4^a^ (0.2)	5.5^a^ (0.2)	5.0^a^ (0.2)	5.3^a^ (0.2)	5.4^a^ (0.2)	5.5^a^ (0.2)

ANOVA, analysis of variance; Co, control; R, ribose; C, carnosine; A, ascorbic acid; RA, ribose + ascorbic acid; RC, ribose + carnosine; RCA, ribose + carnosine + ascorbic acid. Mean values within the same variable “gender,” “age group,” and “formulation” with the same letter within the same row (attribute) are not significantly different with a significance level of (*P* < 0.05).

1 9 = like extremely; 8 = like very much; 7 = like moderately; 6 = like slightly; 5 = neither like nor dislike; 4 = dislike slightly; 3 = dislike moderately; 2 = dislike very much; 1 = dislike extremely.

2 9 = I would eat this every opportunity I had; 8 = I would eat this very often; 7 = I would frequently eat this; 6 = I like this and would eat it now and then; 5 = I would eat this if available but would not go out of my way; 4 = I do not like this but would eat it on an occasion; 3 = I would hardly ever eat this; 2 = I would eat this if there were no other food choices; 1 = I would eat this only if forced.

NS: *P* ≥ 0.05, *P < 0.05, **P < 0.01, ***P < 0.001.

All mean acceptance attribute values for all of the samples were 5.7 and greater, which is within the range of “like slightly” (Table 4). Mean values for eating behavior for all of the samples were between 5.0 and 5.5 which is within the range of “I would eat this if available but would not go out of my way.”

**Figure 2:**
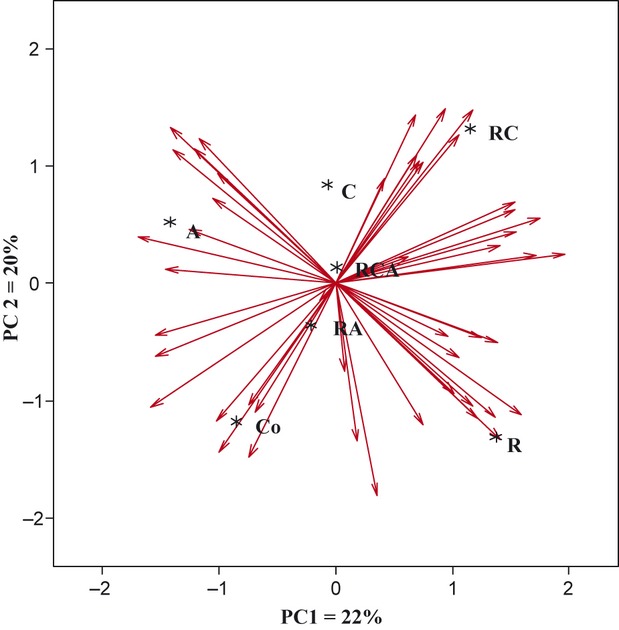
Overall acceptability by consumers (*n* = 59) of seven minced bison formulations. Co, control; R, ribose; C, carnosine; A, ascorbic acid; RA, ribose + ascorbic acid; RC, ribose + carnosine; RCA, ribose + carnosine + ascorbic acid.

Principal component analysis using overall acceptability values for each of the seven samples from each of the 59 consumers determined that 44% of the variability was attributed to the first two principal components. Consumer vectors shown in Figure 2 are spread throughout the plot suggesting that for overall acceptance the consumers were distributed among the samples rather than favoring particular ones.

#### Multiple linear regression analysis

Sensory attribute intensities and all of the physical and chemical measurements were used in the regression model with aroma acceptance as the dependent variable. Cooking loss percentage was the predictor that resulted from the analysis. The associated β-value was −0.437, *P* < 0.048. The stepwise model was significant (*F*_1,19_ = 4.473, *P* < 0.048) with the adjusted *R*^2^ of 0.148. A lower cooking loss resulting in higher moisture could favor an increase in water-soluble compounds responsible for aroma/flavor formation.

#### Partial least squares regression analysis

**Figure 3:**
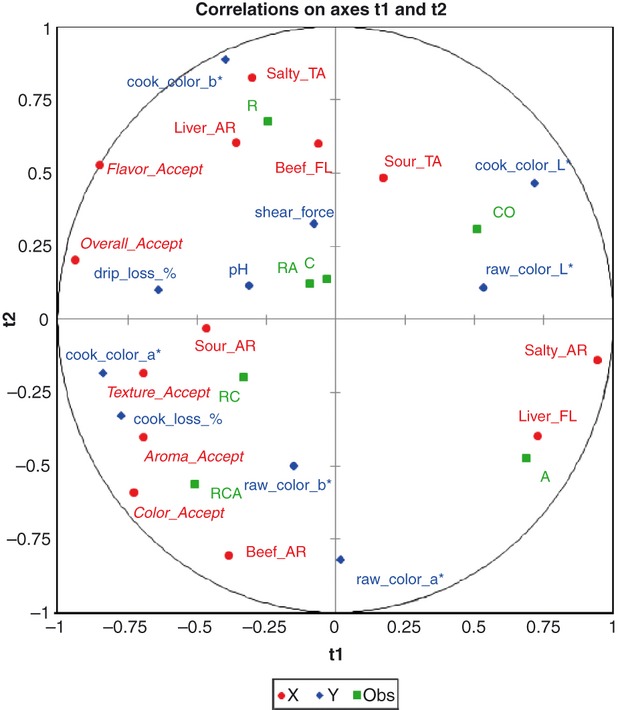
Partial least square (PLS) correlation loadings for *X* – t1 and *Y* – t2, where *X* variables (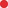) are the sensory components comprised of aroma (AR), flavor (FL), and taste (TA) attributes measured by the trained panel and acceptance (*Accept*) measured by the consumer panel for five parameters – *color*, *aroma*, *flavor*, *texture*, and *overall*, and *Y* variables (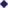) are physical and chemical measurements. Seven formulations (Obs 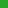) are Co, control; R, ribose; C, carnosine; A, ascorbic acid; RA, ribose + ascorbic acid; RC, ribose + carnosine; RCA, ribose + carnosine + ascorbic acid.

The biplot in Figure 3 shows the correlation between the *x* variables of sensory attributes by the trained and consumer panel and the *y* variables of the physical and chemical measurements. The seven sample observations are also plotted to show the components that characterize them. Samples were separated throughout the biplot indicative of their unique characteristics: the control sample in the upper right, the ascorbic acid sample in the lower right, the RCA and RC samples in the lower left, and the RA, C, and R samples in the upper left portion.

The only sensory attribute shown with Co was sour taste with the L* for both cooked and raw measurements found there as well. Liver flavor and salty aroma were the only sensory attributes associated with A. The a* value for raw color was shown in the same quadrant which is expected as ascorbic acid has been shown to increase the redness in raw meat. None of the acceptance attributes appeared with the Co and A samples inferring a general lower acceptance for these samples.

The ribose sample was situated in the top left with the sensory attributes of liver aroma, beef flavor, and salty taste as well as flavor acceptance and b* for cooked color. RA and C were close to the origin of the biplot where overall acceptability, DL%, pH, and shear force were also found. RC was in the top of the bottom left portion with sour aroma, a* for cooked color, cooking loss%, and texture acceptance. RCA was closer to the bottom of the bottom left portion with aroma acceptance, color acceptance, beef aroma, and b* for color of raw meat in the same area. It would appear that by adding ribose and carnosine individually and in combination with bison patties, consumer acceptance is influenced as well as the majority of the sensory attributes and other quality parameters measured. In terms of the acceptance attributes and measured attributes, flavor and overall acceptance shared the same quadrant with liver aroma, salty taste, and beef flavor. DL%, pH, shear force, and b* for cooked color also appeared in the same quadrant suggesting some relationship between these properties of the bison samples. Texture, aroma, and color acceptance appeared in the bottom left quadrant with sour and beef aromas. Cook color a*, raw color b*, and cooking loss% also appeared in this quadrant.

## Conclusions

The objective of this study was to investigate the synergistic effect of selected ingredients on the flavor, acceptability, and physico-chemical properties of minced bison meat. The RCA sample had significantly higher aroma acceptance compared with Co, C, and RA. This may be related to the high beef aroma intensity found by the descriptive analysis panel as these two variables were found in close proximity on the PLS biplot. Among all of the physico-chemical and sensory attributes, cooking loss percentage was shown from multiple linear regression analysis to have the most influence on aroma acceptance. Although there was no significant formulation effect for color acceptance, RCA had the highest color acceptance value which may be related to the significantly higher a* value for the cooked sample compared with Co, C, and A. RCA also had high overall acceptance with the mean value corresponding to “like slightly.” Further investigation of the synergistic role of ribose, carnosine, and ascorbic acid by increasing the levels and including glucose in minced bison meat may show significant improvements in quality and thus enhance the overall consumer acceptability. Grilling, a method whereby meat is cooked directly on the heated surface, may produce other volatiles that impact the aroma and flavor attributes of bison patties with added ingredients and should be included in future research work. Shelf-life studies would be recommended once the optimum levels of additives are determined.

## References

[b1] Aliani M, Farmer LJ (2005). Precursors of chicken flavor. II. Identification of key flavour precursors using sensory methods. J. Agric. Food Chem.

[b2] Calkins CR, Hodgen JM (2007). A fresh look at meat flavor. Meat Sci.

[b3] Chen Y, Ho C (2002). Effects of carnosine on volatile generation from Maillard reaction of ribose and cysteine. J. Agric. Food Chem.

[b4] Das AK, Anjaneyulu ASR, Biswas S (2006). Effect of carnosine preblending on the quality of ground buffalo meat. Food Chem.

[b5] Decker EA, Crum AD (1991). Inhibition of oxidative rancidity in salted ground pork by carnosine. J. Food Sci.

[b6] Firmage-O'Brien K (2008). http://www.statcan.gc.ca/pub/96-325-x/2007000/article/10504-eng.pdf.

[b7] Ganhão R, Morcuende D, Estévez M (2010). Protein oxidation in emulsified cooked burger patties with added fruit extracts: influence on colour and texture deterioration during chilled storage. Meat Sci.

[b8] Health Canada (2010). http://webprod3.hc-sc.gc.ca/cnf-fce/start-debuter.do?lang=eng.

[b9] Joseph P, Suman SP, Li S, Beach CM, Steinke L, Fontaine M (2010). Characterization of bison (*Bison bison*) myoglobin. Meat Sci.

[b10] Kansci G, Genot C, Meynier A, Gandemer G (1997). The antioxidant activity of carnosine and its consequences on the volatile profiles of liposomes during iron/ascorbate induced phospholipid oxidation. Food Chem.

[b11] Lee BJ, Hendricks DG, Cornforth DP (1999). A comparison of carnosine and ascorbic acid on color and stability in a ground beef pattie model system. Meat Sci.

[b12] Li Q, Logue CM (2005). The growth and survival of *Escherichia coli* O157:H7 on minced bison and pieces of bison meat stored at 5 and 10°C. Food Microbiol.

[b13] Lundahl DS, McDaniel MR (1988). The panelist effect – fixed or random?. J. Sens. Stud.

[b14] MacFie HJH, Thomson DMH, Piggott JR (1988). Multidimensional scaling methods. Sensory analysis of foods.

[b15] MacLeod G, Shahidi F (1994). The flavor of beef. The flavor of meat and meat products.

[b16] Manley CH, Choudhury BH, Mazeiko P, Ashurst PR (1999). Thermal process flavorings. Food flavorings.

[b17] McAfee AJ, McSorley EM, Cuskelly GJ, Moss BW, Wallace JMW, Bonham MP (2010). Red meat consumption: an overview of the risks and benefits. Meat Sci.

[b18] McClenahan JM, Hamouz FL, Setiawan B, Marchello MJ, Driskell JA (2001). Sensory evaluation of broiled and grilled bison patties by trained panelists. J. Food Qual.

[b19] Meinert L, Schafer A, Bjergegaard C, Aaslyng MD, Bredie WLP (2009). Comparison of glucose, glucose 6-phosphate, ribose, and mannose as flavor precursors in pork; the effect of monosaccharide addition on flavor generation. Meat Sci.

[b20] Mottram DS, Maarse H (1991). Meat. Volatile compounds in foods and beverages.

[b21] National Bison Association (2012). http://www.bisoncentral.com/about-bison/data-and-statistics.

[b22] O'Mahony M (1986). Sensory evaluation of food: statistical methods and procedures.

[b23] Sanderson K, Hobbs JE, Shand P, Kerr W (2003). Consumer preferences in the emerging bison industry. J. Int. Food Agribus. Mark.

[b24] SAS Statistical System (2003). SAS OnlineDoc®.

[b25] Sato K, Hegarty GR (1971). Warmed-over flavor in cooked meats. J. Food Sci.

[b26] Schutz HG (1965). A food action rating scale for measuring food acceptance. J. Food Sci.

[b27] Sensory Information Management System (2011). SIMS 2000, Version 6.

[b28] SPSS Statistics (2010). Statistical Package for the Social Sciences (Version 19.0).

[b29] Stone H, Sidel JL (2004). Sensory evaluation practices.

[b30] Troutt ES, Hunt MC, Johnson DE, Claus JR, Kastner CL, Kropf DH (1992). Chemical, physical and sensory characterization of ground beef containing 5 to 30 percent fat. J. Food Sci.

[b31] XLSTAT (2012). Running a partial least squares (PLS) discriminant analysis with XLSTAT-PLS. http://www.xlstat.com/en/learning-center/tutorials/running-a-partial-least-square-pls-discriminant-analysis-with-xlstat-pls.html#.

[b32] Yuan X, Marchello MJ, Driskell JA (1999). Selected vitamin contents and retentions in bison patties as related to cooking method. J. Food Sci.

